# Supplementary material for: Constructing Qubit Edge States by Inverse-Designing the Electromagnetic Environment
Source: ACS Photonics. 2025 Sep 25;12(10):5434–42. doi: 10.1021/acsphotonics.5c00986 (PMC12532289; doi:10.1021/acsphotonics.5c00986)
Supplement: Supplementary file 1 [file ph5c00986_si_001.pdf]

Supporting Information: Constructing qubit edge states by inverse-designing the electromagnetic environment

A. Miguel-Torcal,* T. F. Allard, P. A. Huidobro, F. J. García-Vidal, and A. I. Fernández-Domínguez*

Departamento de Física Teórica de la Materia Condensada, Universidad Autónoma de Madrid, E- 28049 Madrid, Spain.

Condensed Matter Physics Center (IFIMAC), Universidad Autónoma de Madrid, E- 28049 Madrid, Spain.

E-mail: alberto.miguel@uam.es; a.fernandez-dominguez@uam.es

Abstract

This Supporting Information explores an alternative optimization strategy for engineering topological phases in our qubit chain by simultaneously tuning coherent and dissipative coupling parameters through a unified target function. Unlike the original two-stage procedure—where coherent and dissipative terms were optimized sequentially—this single-stage approach converges to a stable configuration of interaction parameters but yields reduced performance. Specifically, it leads to stronger chiral symmetry breaking, diminished edge-bulk decoupling, and lower robustness of edge states in the presence of disorder. Although the inverse-designed structure still sup-

ports topological features, it shows greater sensitivity to imperfections, suggesting that the increased constraints of the unified objective function hinder optimal realization.

1 One-stage optimization process

The optimization procedure, as detailed in the main text, was carried out in two sequential stages. Initially, we adjusted the coherent coupling strength parameters that govern the dipole-dipole interaction, while allowing the parameters responsible for radiative decay and dissipative coupling to vary freely. In the second stage, this configuration was reversed. Here, we present results from an alternative approach where both coherent and dissipative parameters were optimized simultaneously by merging the two target functions into a single unified one, which reads $f = |J_{AB}|/|J_{BA}| \times |J_{AA}| \times |J_{BB}| \times |\gamma_{AB}| \times |\gamma_{BA}|$. Although this combined optimization may appear more straightforward, it proves to be less efficient in practice. Specifically, it leads to a more pronounced breaking of chiral symmetry, reduced decoupling between edge and bulk states dynamics, and diminished robustness of the edge modes in the presence of disorder.

Fig. S1 displays five panels that replicate Figs. 1, 2, and 4 in the main text, now corresponding to the parameters obtained from the single-stage optimization procedure. Figs. S1(a) and S1(b) depict the coherent and dissipative coupling strength parameters involved in the target function f, along with the second-nearest-neighbor dissipative interaction coefficients, γ_{AA} and γ_{BB}, normalized by the collective decay rate $\gamma = \sqrt{\gamma_A \gamma_B}$. The insets in each panel, respectively, show the ratio $|J_{AB}|/|J_{BA}|$ and the individual radiative decay rates of the qubits within the unit cell. The curves appear to follow similar trajectories toward configurations that support the intended topological phase and the emergence of edge states in the qubit chain. However, they exhibit notable differences compared to those obtained via the two-stage optimization approach. Notably, all the parameters represented now converge to static values over the final 150 iterations, indicating that the optimization has approached

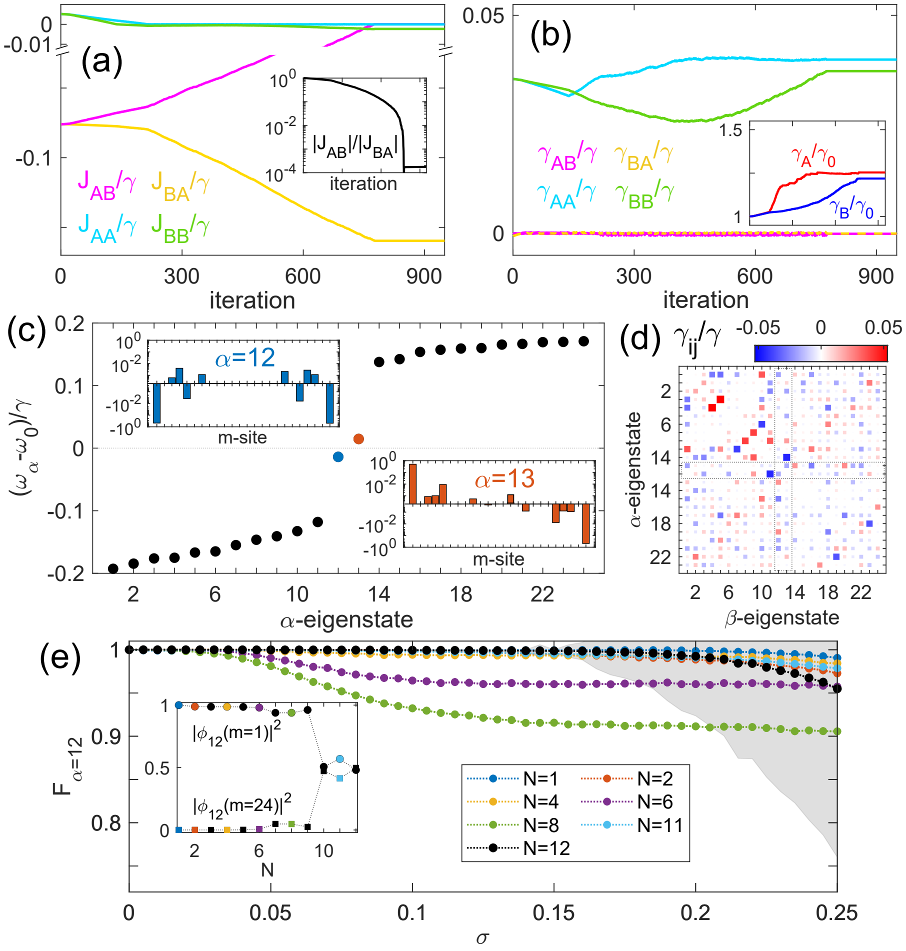

Figure S1: Single-stage optimization performance. (a) Qubit-qubit coherent interaction strengths as a function of the iteration step. Inset: Coupling strength ratio between nearest neighbors inside and outside the unit cell. (b) First and second nearest neighbors dissipative coupling parameters versus iterations. Inset: Decay rate normalized to free space radiation of each of the qubits inside the unit cell. (c) Eigenstates of the Hamiltonian within the single excitation subspace. Insets: Real-valued wavefunction of the two edge-states (indicated with blue and red circles) in log scale, $\pm\text{sign}(\phi_\alpha)|\phi_\alpha|^2$ ($\alpha = 12, 13$). (d) Dissipative matrix in the Hamiltonian eigenstates basis normalized to the collective decay rate $\gamma = \sqrt{\gamma_A \gamma_B}$. The diagonal terms (which correspond to the eigenstate decay rates) are not shown. (e) Robustness of the edge state ϕ_{12} against normally distributed random noise introduced in the coherent coupling strengths and for different spatial ranges of the qubit-qubit interactions, indicated by N. F_α is the fidelity between disordered and non-disordered edge state. Inset: Population of the edge-state ϕ_{12} at the chain's boundary sites.

a configuration where no further modification of the dielectric permittivity distribution can enhance the unified target function. This suggests that the increased restrictiveness of the single-stage target function has guided the system toward a near-optimal combination of

interaction parameters.

A particularly striking feature is that both the intra- and inter-cell dissipative coupling strengths between nearest neighbors remain nearly negligible throughout the optimization. This behavior is dictated by the constraints inherently imposed by the unified target function from the start of the optimization. Additionally, the ratio $|J_{AB}|/|J_{BA}|$ reaches a minimum that is few orders of magnitude lower than its counterpart in the two-stage strategy, while the normalized individual decay rates, γ_A/γ_0 and γ_B/γ_0, attain similar values. This suggests that the inverse-designed cavity (truncated waveguide) yields coupling parameters that are more aligned with those required for realizing a nontrivial phase in the SSH model. However, the inter-cell coherent interaction rates connecting sites of the same character (either A or B) exceed the intra-cell nearest-neighbor coupling rates, i.e. $|J_{BB}| > |J_{AA}| \sim |J_{AB}|$ (see Fig. S1(a)), deviating from the conventional configuration of extended (long-range) SSH-like systems. This imbalance constitutes a noticeable manifestation of chiral symmetry breaking. As a direct consequence, the edge state eigenenergies undergo splitting, pushing them farther apart. This effect is evident in the colored markers in Fig. S1(c), which display the eigenvalues—arranged in ascending order—for edge and bulk states, obtained by diagonalizing the Hamiltonian in Equation (2) of the main text. The insets in Fig. S1(c) depict the spatial population distribution of the corresponding edge states across the qubit sites.

Fig. S1(d) reproduces the results shown in Fig. 2(b) in the main text, now based on the optimal structure and parameters obtained from the single-stage optimization. As highlighted by the dashed rectangles, the two edge states exhibit lower mutual population exchange while interacting more prominently with the remaining eigenstates of the system, indicating a less effective dynamical decoupling of the edge modes from the bulk. Finally, the topological character of the engineered system obtained via the single-stage optimization approach is tested in the presence of noise and disorder. The sensitivity and degree of protection of the resulting edge states are assessed by introducing random noise to simulate

potential defects or imperfections in the chain. The fidelity between the edge states of the disordered and non-disordered systems is then computed, incorporating the cut-off imposed on the spatial range of the coherent coupling parameters. Fig. S1(e) shows the fidelity as a function of the normalized standard deviation, σ, for each interaction range considered. A comparison with Fig. 4(b) in the main text reveals that, even though the edge states still demonstrate notable robustness—maintaining over 90% fidelity—the dielectric structure resulting from the single-stage optimization exhibits increased fragility under growing disorder levels.
